# H_2_S Donor SPRC Ameliorates Ischemic Stroke by Upregulating CD24

**DOI:** 10.1111/cns.70243

**Published:** 2025-02-15

**Authors:** Chenye Wang, Sha Li, Qixiu Li, Haiyan Xi, Jiejia Li, Qing Zhu, Pinwen Wu, Yi‐Zhun Zhu, Yicheng Mao

**Affiliations:** ^1^ Department of Pharmacology, the Key Laboratory of Smart Drug Delivery (Ministry of Education), School of Pharmacy Minhang Hospital Fudan University Shanghai China; ^2^ School of Pharmacy and Laboratory of Drug Discovery from Natural Resources and Industrializtion Macau University of Science and Technology Macau China; ^3^ School of Pharmacy Provincial Key Laboratory of Inflammation and Molecular Drug Target Institute for Translational Neuroscience Affiliated Hospital 2 of Nantong University Centre for Neural Developmental and Degenerative Research Nantong University Nantong China

**Keywords:** CD24, H_2_S, ischemic stroke, neuroinflammation, SPRC

## Abstract

**Background:**

Ischemic stroke is well‐known for its high mortality and morbidity, but its treatment remains to be explored due to the current limitations. For example, severe neuroinflammation occurs after ischemic stroke; however, effective neuroinflammatory inhibitors are still lacking. Thus, the development of new therapeutic targets of inhibiting neuroinflammation is urgent. CD24 is a small heavy glycosylated protein, which plays a critical role in neural development and acts as an inflammatory suppressor in tumors and autoimmune diseases. But the role of CD24 in ischemic stroke remains unknown.

**Aims:**

The role of CD24 in ischemic stroke should be explored. Additionally, the potential relationship between the H_2_S donor, S‐propargyl‐cysteine (SPRC) and CD24 in ischemic stroke should be revealed.

**Methods:**

Mechanism studies have been performed both in vitro and in vivo to verify the CD24‐mediated inflammation and migration. SPRC has been applied to treat ischemic stroke, and its potential association with CD24 has been studied.

**Results:**

The overexpression of CD24 can inhibit the nuclear factor kappa B (NF‐κB) inflammatory signaling pathway and promote the migration ability of M2 microglia cells via Src/Fak/Pyk2 signaling pathway in an inflammatory model of BV2 cells. SPRC can upregulate the level of endogenous H_2_S via cystathionase‐β‐synthase (CBS) and it indirectly plays a role in upregulating CD24.

**Conclusions:**

CD24 could be a potential target of inhibiting neuroinflammation. SPRC reduces inflammation in ischemic stroke by regulating the CD24/Iκ‐Bα/NF‐κB inflammatory signaling pathway and improves the migration ability of M2 microglia via CD24/Src/Fak/Pyk2 signaling pathway, which further alleviates the inflammatory response at the lesion.

## Graphical abstract

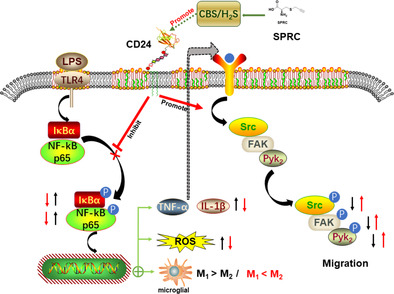

## 1 Introduction

Ischemic stroke and its sequelae represent the leading cause of morbidity and mortality [1]. The treatments of ischemic stroke mainly include surgical therapies, thrombolytic drugs, anticoagulant drugs, fibrinolytic drugs, antiplatelet aggregation drugs, etc. [2, 3]. But there are some limitations to these approaches [2, 3, 4, 5, 6]. The pathogenesis of ischemic stroke is complex and every factor influences each other, so it remains necessary to develop new therapies for ischemic stroke based on different pathogenesis [7]. Neuroinflammation is an important pathogenesis of ischemic stroke [8, 9]. When ischemic stroke occurs, neuroinflammation in the brain breaks out rapidly, and microglia cells are central to the repair of tissue damage under pathological conditions [8, 10, 11]. However, there are few effective neuroinflammatory inhibitors [5]. Thus, the development of new therapeutic targets for inhibiting neuroinflammation is urgent in ischemic stroke.

CD24 is known as a heat‐stable antigen or small‐cell lung carcinoma cluster 4 antigen [12, 13, 14]. It is also called a heavily glycosylated glycosylphosphatidylinositol (GPI)‐anchored surface protein [12, 15]. CD24 is widely distributed in hematopoietic and neuronal cells, many tissues, and some tumor stem cells, so it has been extensively used as a marker for adaptive immunity, inflammation, autoimmune diseases and cancers [15, 16, 17]. It has been revealed that CD24 is central to cellular communication and cell functions, including neural migration, neurite outgrowth and neurogenesis, just like other neural cell surface antigens [18]. Li et al. [19] have indicated that CD24 expression could negatively regulate the NF‐κB/inflammatory factor pathway after experimental TBI in mice, which providing a novel target for the therapeutic intervention of TBI. Wang et al. [20] identified the CD24‐Siglec axis as an innate immune checkpoint against metaflammation and metabolic disorder, suggesting a promising therapeutic target for metabolic diseases. More and more studies have shown that CD24 is a new target for anti‐inflammation, which has certain clinical significance. So CD24 may be also a very potential neuroprotective agent of ischemic stroke.

H_2_S is now widely recognized as an important endogenous signaling molecule in mammalian cells and tissues, produced by three principal mammalian enzymes: cystathionine‐β‐synthase (CBS), cystathionine‐γ‐lyase (CSE), and 3‐mercaptopyruvate sulfurtransferase (3‐MST) [21, 22]. H_2_S plays an important role in the central nervous system and cardiovascular system, such as anti‐oxidation, anti‐apoptosis, anti‐inflammation, anti‐fibrosis, and so on [22, 23, 24, 25, 26, 27]. SPRC is a structural analog of S‐allylcysteine (SAC), a plentiful constituent of garlic extract. Our previous studies have reported that SPRC has good effects on relieving inflammation on cardiovascular disease and autoimmune disease through elevating H_2_S level by modulating CSE/H_2_S pathway [28, 29, 30, 31]. However, the role of hydrogen sulfide plays in stroke remains controversial. The current evidence suggests that the presence of H_2_S in the ischemic brain may be either harmful or protective depending on its concentration, harmful when high and protective when low [32, 33]. In view of these, the treatment of SPRC in ischemic stroke associated with its regulation ability of H_2_S needs to be further studied.

Here, to explore the role of CD24 that plays in ischemic stroke, mechanism studies have been performed both in vitro and in vivo to verify the CD24‐mediated inflammation and migration. Additionally, SPRC has been used to treat ischemic stroke, and its potential relationship with CD24 has been investigated.

## 2 Materials and Methods

### 2.1 Animals

SPF level male C57BL/6J mice, weight 20 ± 2 g, and age 8 weeks, were purchased from Shanghai Lingchang Biotechnology. All animal experimental protocols were approved by the Animal Care Committee of Fudan University and conformed to Journal Pre‐proof the Animal Management Rules of the Ministry of Health of the People's Republic of China.

### 2.2 Establishment of an Animal Model of Middle Cerebral Artery Occlusion (MCAO) and Drug Treatment

The mice were randomly divided into four groups, seven mice in each group. Sham groups (Sham): Mice received surgery without MCAO; MCAO group (MCAO): Mice were subjected to MCAO; treatment group (MCAO + SPRC): Intraperitoneal injection SPRC (10 mg/kg) was performed immediately after modeling, and then once every 12 h for three times; CBS Inhibitor group (MCAO + SPRC + AOAA): the CBS inhibitor AOAA (10 mg/kg) was intraperitoneally injected 2 h before modeling. After modeling, SPRC (10 mg/kg) was administered by intraperitoneal injection, then once every 12 h for three times. After 48 h, brain tissues were extracted. Mice were anesthetized with 4% chloral hydrate (10 μL/g) by intraperitoneal injection. The mice were fixed to a board after full anesthesia. The MCAO model of the left brain of the mice was made under a microscope. Surgical separation of the right common carotid artery (CCA), internal carotid artery (ICA), and external carotid artery (ECA). The ECA was ligated, and a filament with a silicon tip was then inserted through the ECA stump into the ICA until resistance was felt, occluding the middle cerebral artery. After 1 h of MCAO, the suture was removed and the blood circulation was recovered. The mice were put back into a warm container at 37°C to recover. Injecting with 0.9% NaCl and 10% glucose after surgery to reduce the mortality and monitoring the vital signs of mice during and after surgery.

### 2.3 Cell Cultures

BV2 cells are mouse microglia (immortalized). The cell line was a gift of Chinese Academy of Sciences. BV2 cells were cultured in DMEM medium containing 5.5 mM glucose supplemented with 10% FBS and 1% penicillin/streptomycin at 37°C in humidified 5% CO_2_ conditions. Trypsin–EDTA was used to passage the cells. When the cell density reached 80%–90%, the passage was carried out in a ratio of 1:2–1:4. The storage medium of BV2 cells was 95% FBS with 5% DMSO.

### 2.4 Lipopolysaccharide (LPS)‐Induced Inflammatory Model of BV2 Cells

Well‐grown cells were seeded in to 6‐well plates, and cell transfection was performed when the cell density was 60%. The overexpression plasmid of pcDNA3.1(+), pcDNA3.1(+)‐CD24 (Sangon Biotech, China) and the small interfering RNA of siRNA NC, siRNA CD24 (RIB BIO, China) were transfected into BV2 cells using Lipo8000 Transfection Reagent (Beyotime Biotechnology, China) following the protocol stipulated by the manufacture. After 24 h, BV2 cells were stimulated with 1 μg/mL LPS cell culture medium for 24 h and then mRNA and protein were harvested.

### 2.5 Double Immunofluorescent Staining

The mice were anesthetized and fixed to a board. The chest of the mice was cut open, exposing the heart and liver. A needle was inserted into the left ventricle of a mouse and the liver was cut up with scissors. First, 0.9% NaCl was slowly injected to flush the blood until the limbs, liver, and tongue turned white. Then 60 mL 4% paraformaldehyde was slowly infused to fix the brain tissue. During the infusion of paraformaldehyde, the tail of mice may have a reflex phenomenon. Then the brain tissue was extracted and placed in a tube containing 4% paraformaldehyde, marked, and stored in a refrigerator at 4°C. The brain tissue was then sent to Servicebio Co. Ltd. for embedding and frozen section treatment, and complete the double immunofluorescent staining.

### 2.6 Quantitative Real‐Time Polymerase Chain Reaction (qRT‐PCR) Analysis

Total RNA was isolated from cells using TRIzol Reagent (ABclonal, China) according to the manufacturer's protocol, quantified using NanoDrop spectrophotometer (Thermo Scientific, USA), and reverse transcription into cDNA was conducted by CFX Connect Real‐time System (Bio‐Rad, USA). Expression of target genes was measured by semiquantitative qPCR using Hieff UNICON qPCR SYBR Green Master Mix (ABclonal, China). *Gapdh* was used as the reference gene for analyzing. Relative target gene expression was analyzed by the 2^−△△Ct^ method. The gene‐specific sequences used in this study are shown in Table 1.

**TABLE 1:** Primer sequences were used in this study.

Genes	Forward and reserve primers (5′ → 3′)
Mouse *Gapdh*	F: GTTTCCTCGTCCCGTAGACA
	R: GATGGGCTTCCCGTTGATGA
Mouse *Cd24*	F: TTCTGGCACTGCTCCTACC
	R: GCGTTACTTGGATTTGGGGAA
Mouse *Il1b*	F: CTCGTGCTGTCGGACCCAT
	R: CAGGCTTGTGCTCTGCTTGTGA
Mouse *Tnfa*	F: ATGAGCACAGAAAGCATGATC
	R: TACAGGCTTGTCACTCGAATT
Mouse *Il6*	F: TCTATACCACTTCACAAGTCGGA
	R: GAATTGCCATTGCACAACTCTTT
Mouse *Cd68*	F: TGTCTGATCTTGCTAGGACCG
	R: AGGAGAGTAACGGCCTTTTTG
Mouse *Cd163*	F: CCAAGCTGTGAAGGCACTAAA
	R: ACGGTTTGGCAGGACAATC
Mouse *Cd206*	F: GGATTGTGGAGCAGATGGAAG
	R: CTTGAATGGAAATGCACAGA
Mouse *Il10*	F: CAGTGGAGCAGGTGAAGAGTGA
	R: CCTGGAGTCCAGCAGACTCAAT

### 2.7 Western Blotting

Proteins were lysed in RIPA Lysis Buffer (Beyotime Biotechnology, China), and the concentrations were determined using the bicinchoninic acid assay. Protein samples were fractionated using sodium dodecyl sulfate (SDS)‐polyacrylamide gel electrophoresis and transferred to PVDF membranes. Following blocking for 1 h with 5% skim milk in Tris Buffered saline Tween (TBST), membranes were incubated with antibodies including CD24 (1:1000, ABclonal, China), IL‐1β (1:1000, Santa, USA), TNF‐α (1:1000, Proteintech, China), Iκ‐Bα (1:10000, Abcam, UK), p‐Iκ‐Bα (1:1000, ABclonal, China), NF‐κB p65(1:10000, Abcam, UK), p‐NF‐κB p65 (1:1000, ABclonal, China), c‐Src (1:10000, Abcam, UK), p‐cSrc (1:1000, Santa, USA), Fak(1:1000, CST, USA), p‐Fak(1:1000, ABclonal, China), Pyk2 (1:1000, ABclonal, China), p‐Pyk2 (1:1000, ABclonal, China), CD206 (1:1000, ABclonal, China), CD86 (1:1000, ABclonal, China), CBS (1:1000, Proteintech, China), β‐tubulin(1:10000, Proteintech, China), β‐actin (1:1000, Santa, USA) and GAPDH (1:10000, Proteintech, China) overnight at 4°C. After being washed in TBST, membranes were incubated with HRP‐conjugated secondary antibody at room temperature for 1 h, and protein was detected using an Immobilon Western HRP. Signal intensities were quantified by gel imaging system (ChemiDoc XRS, Bio‐Rad, USA). Quantification of blots was performed by Image Lab.

### 2.8 Cell Migration

Using a marker pen to draw parallel lines on the underside of the 6‐well plate. When the density of cells in the 6‐well plate was up to 100% using a spear to make cell scratches in a direction perpendicular to the lines marked before. The floating cells were washed to be removed with PBS for three times. Then serum‐free medium was added to the 6‐well plate to continue the cell culture, and photos were taken at intervals using an inverted microscope. The cells were washed thrice with PBS before each photo shoot. After taking photos each time, put the cells to the incubator for further cultivation. The areas of scratches were analyzed using ImageJ.

### 2.9 Reactive Oxygen Species (ROS) Analysis

2′,7′‐Dichlorodihydrofluorescein diacetate (DCFH‐DA) was diluted in serum‐free medium at 1:1000 to achieve a final concentration of 10 μmol/L. The cell culture medium was removed, and an appropriate volume of diluted DCFH‐DA was added. After incubating at 37°C for 20 min, the cells were washed three times with serum‐free cell culture solution to remove the DCFH‐DA that did not enter the cells. The cells were detected with confocal scanning microscopy.

### 2.10 Triphenyl Tetrazolium Chloride (TTC) Staining

Fresh mouse brain tissue was rapidly frozen at −20°C for 5 min, and continuous coronal cutting was performed with a blade at a 2 mm interval. The slices were placed in 0.2% TTC solution and incubated for 30 min at 37°C away from light. Samples were fixed with 4% paraformaldehyde overnight. TTC can stain normal brain tissue red and infarct areas white.

### 2.11 H_2_S Concentration in Serum Analysis

Fresh blood was collected and maintained at room temperature for 1–2 h, centrifuged at 6000 rpm at 4°C for 10 min, and then the supernatant (serum) was taken. H_2_S of serum content of mice was detected with a Micro H_2_S Content Assay Kit (Solarbio, Beijing) according to the manufacturer's protocol.

### 2.12 Statistical Analysis

All the data were analyzed and performed by Prism software 8.0 (GraphPad Software Inc., San Diego, CA, USA) and were shown as the mean ± SEM. When analyzing the differences among three or more groups, one‐way ANOVA (and nonparametric or mixed) was used. For the two groups' analysis, the statistical significance was determined by the *t*‐tests (and nonparametric tests). The difference will be considered statistically significant at the *p* < 0.05 level.

## 3 Results

### 3.1 The Distribution of CD24 and the Expression of CD24 as Well as Pro‐Inflammatory Factors IL‐1β and TNF‐α, in MCAO Mice

**FIGURE 1:**
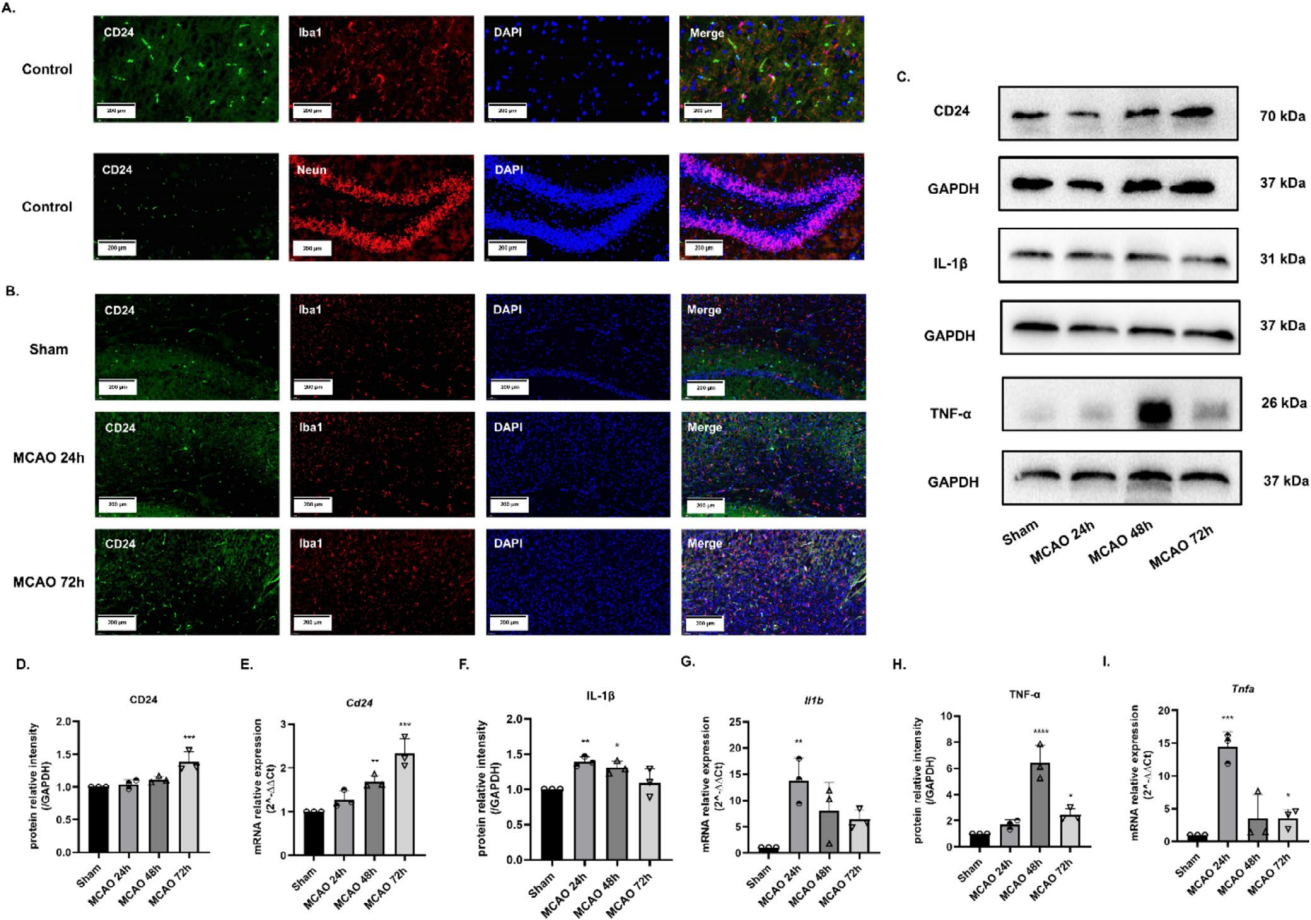
The distribution of CD24 and the expression of CD24 and pro‐inflammatory factors in the brain of MCAO mice. (A, B) Double immunofluorescent staining detected the distribution of CD24 in mice brain. (C–I) WB and qRT–PCR were used to detect the protein and gene expression levels of CD24, IL‐1β, and TNF‐α. All data were expressed as mean ± SEM (*p < 0.05, **p < 0.01, ***p < 0.001 vs. Sham; one‐way ANOVA, *n* = 3).

To demonstrate the distribution of CD24 in mice brain, double immunofluorescent staining of CD24/Iba1 and CD24/Neun was performed. The results showed that CD24 was mainly distributed in microglia cells but less in hippocampal neurons (Figure 1A). Then, we divided mice into four groups according to different reperfusion times: Sham, MCAO 24 h, MCAO 48 h, and MCAO 72 h. Immunofluorescent staining of CD24/Iba1 results showed that the expression of CD24 in microglia increased with the ascending reperfusion time (Figure 1B). The results of WB and qRT–PCR showed that the expression of CD24 increased with the increasing ischemia–reperfusion time and reached a maximum at 72 h. The expression of pro‐inflammatory factors IL‐1β and TNF‐α was initially increased and then decreased, the levels of pro‐inflammatory factors were significantly decreased 72 h after reperfusion (Figure 1C–I). Therefore, the preliminary assumption was made that CD24 should have a negative regulation of pro‐inflammatory factors, alleviating the inflammatory response in the body.

### 3.2 Overexpression of CD24 Improved the Inflammatory Response of BV2 Cells

**FIGURE 2:**
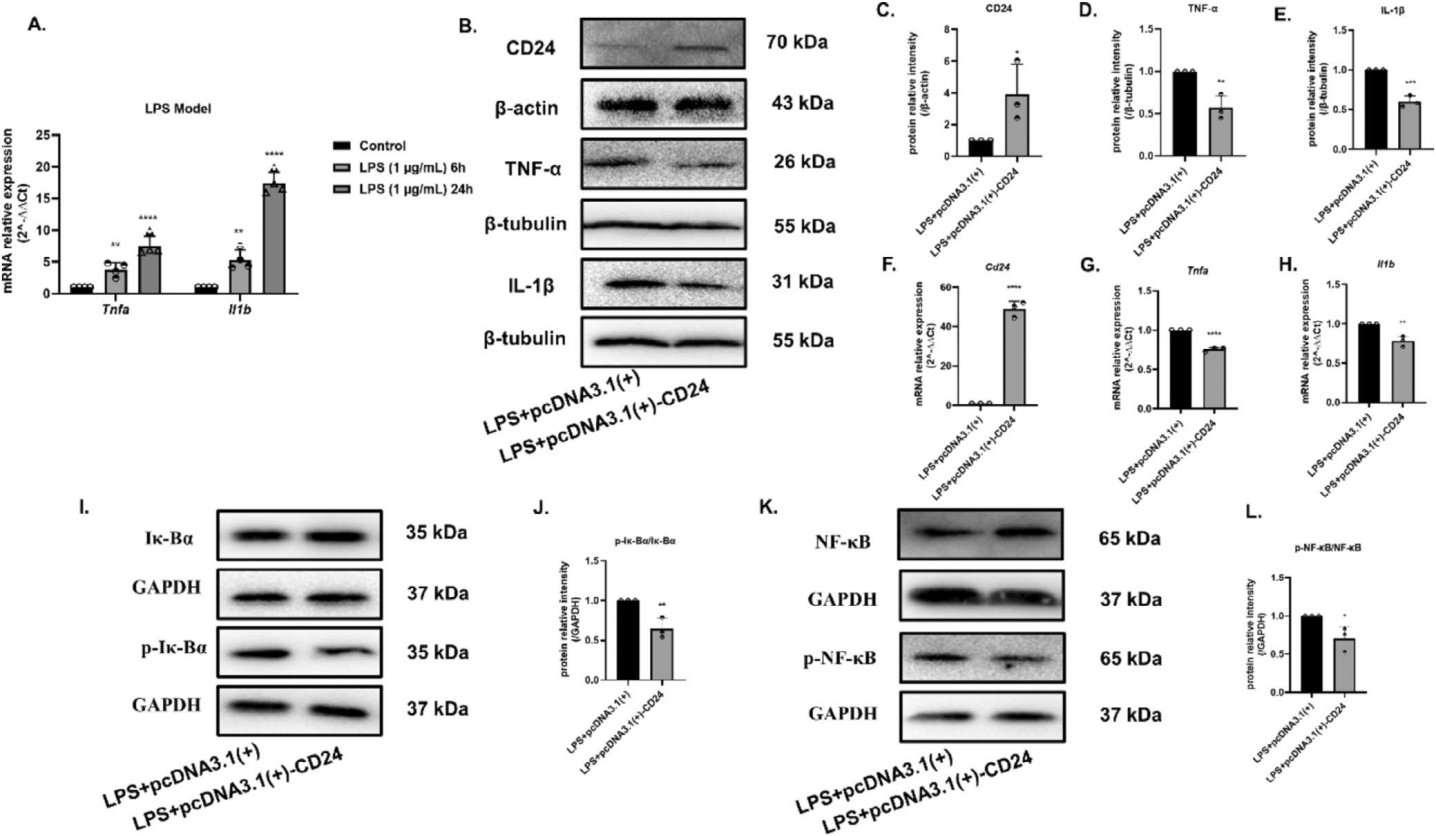
Overexpression of CD24 improved the inflammatory response of BV2 cells via Iκ‐Bα/NF‐κB signaling pathway. (A) The expression levels of LPS‐induced pro‐inflammatory factors were detected by qRT–PCR. (B–H) The expression levels of CD24, TNF‐α, and IL‐1β were detected by WB and qRT–PCR. (I–L) The phosphorylation levels of Iκ‐Bα and NF‐κB p65 were detected by WB. All data were expressed as mean ± SEM (*p < 0.05, **p < 0.01, ***p < 0.001 vs. LPS + pcDNA3.1(+); *t*‐test; *n* = 3).

To further explore CD24‐mediated inflammatory mechanisms, we selected BV2 cells for further investigation. Firstly, we chose 1 μg/mL LPS to induce BV2 cells for 24 h as the modeling condition of neuroinflammation (Figure 2A). Overexpression plasmid pcDNA3.1(+) and pcDNA3.1(+)‐CD24 were used for transfect into BV2 cells through Lipo8000 liposomes. The protein and gene expression levels of CD24, TNF‐α, IL‐1β in BV2 cells were detected by WB and qRT–PCR. The results showed that after transfection with the overexpressed plasmid CD24, the expression of CD24 significantly increased, but the expression of pro‐inflammatory factors TNF‐α, IL‐1β decreased (Figure 2B–H). Therefore, it provides hints that CD24 may negatively regulate pro‐inflammatory factors, playing a role in reducing inflammation. Then, the phosphorylation levels of Iκ‐Bα and NF‐κB p65 were detected, because they can affect the level of pro‐inflammatory factors. The results revealed that after CD24 overexpression, the phosphorylation levels of Iκ‐Bα and NF‐κB were downregulated (Figure 2I–L), further explaining the downregulation effects of pro‐inflammatory factors caused by CD24 in Figure 2B–H.

### 3.3 Overexpression of CD24 Alleviated Oxidative Stress in BV2 Cells and Promoted Microglia to Polarize M2 Type

**FIGURE 3:**
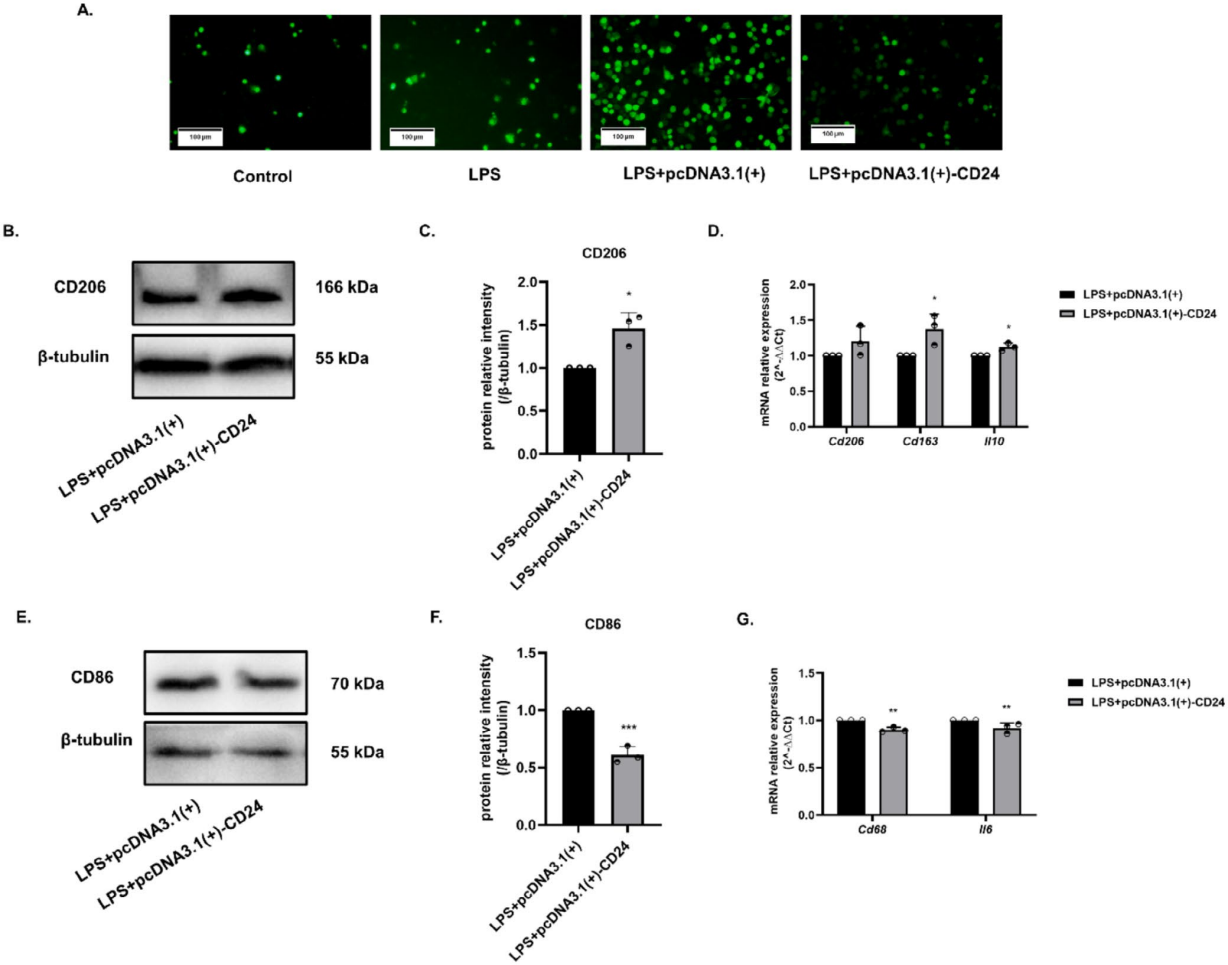
Overexpression of CD24 alleviated oxidative stress in BV2 cells and promoted microglia to polarize M2 type. (A) ROS levels were detected by fluorescence. (B, C) The expression level of CD206 was detected by WB. (D) qRT–PCR was used to detect the gene expression levels of *Cd206, Cd163, and Il10*. (E, F) WB was used to detect the expression level of CD86. (G) qRT–PCR was used to detect the gene expression levels of *Cd68* and *Il6*. All data were expressed as mean ± SEM (*p < 0.05, **p < 0.01, ***p < 0.001 vs. LPS + pcDNA3.1(+); *t*‐test; *n* = 3).

The level of intracellular ROS significantly decreased in cells when CD24 was overexpressed, which indicated that CD24 played a negative regulatory role in the level of oxidative stress (Figure 3A). To prove the hypothesis that CD24 might affect the polarization of BV2, the markers associated with M1 and M2 microglia were detected. The results showed that overexpression of CD24 could increase the expression of CD206, the marker of M2 microglia, and the related molecules *Cd163* and *Il10* also showed with increasing trends (Figure 3B–D). In contrast, the expression level of CD86, the marker of M1 microglia, was downregulated, and its related molecules *Cd68* and *Il6* also showed with downward trend (Figure 3E–G).

### 3.4 Inhibition of CD24 Intensified the Inflammatory Response of BV2 Cells

**FIGURE 4:**
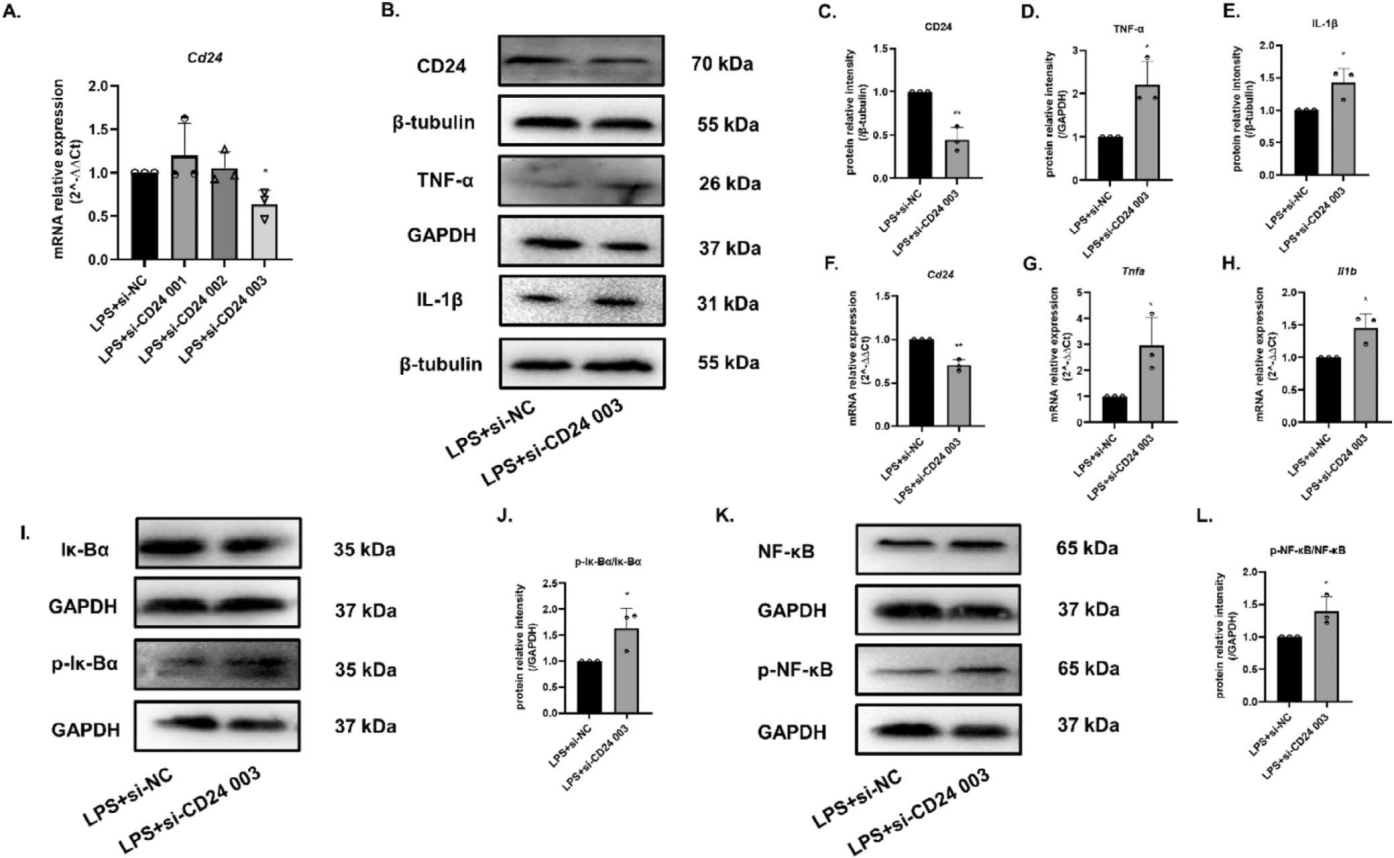
Inhibition of CD24 intensified the inflammatory response of BV2 cells via Iκ‐Bα/NF‐κB signaling pathway. (A) Screening of CD24 small interfering plasmid. (B–H) The expression levels of CD24, TNF‐α, and IL‐1β were detected by WB and qRT–PCR. (I–L) The phosphorylation levels of Iκ‐Bα and NF‐κB p65 were detected by WB. All data were expressed as mean ± SEM (*p < 0.05, **p < 0.01, ***p < 0.001 vs. LPS + LPS + si‐NC; *t*‐test; *n* = 3).

To further verify the CD24‐mediated inflammatory mechanisms, small interfering plasmids were selected to demonstrate the effect of CD24 from the opposite side. According to the results, siRNA CD24 003 had the best knockdown effect (Figure 4A). Therefore, siRNA CD24 003 was selected for subsequent small interference‐related experiments. In contrast, the results showed that with the decreasing expression of CD24, levels of pro‐inflammatory factors TNF‐α and IL‐1β showed the upward trends (Figure 4B–H), and the phosphorylation of Iκ‐Bα and NF‐κB was also up‐regulated (Figure 4I–L).

### 3.5 Inhibition of CD24 Enhanced Oxidative Stress in BV2 Cells and Promoted the Expression of M1 Microglia

**FIGURE 5:**
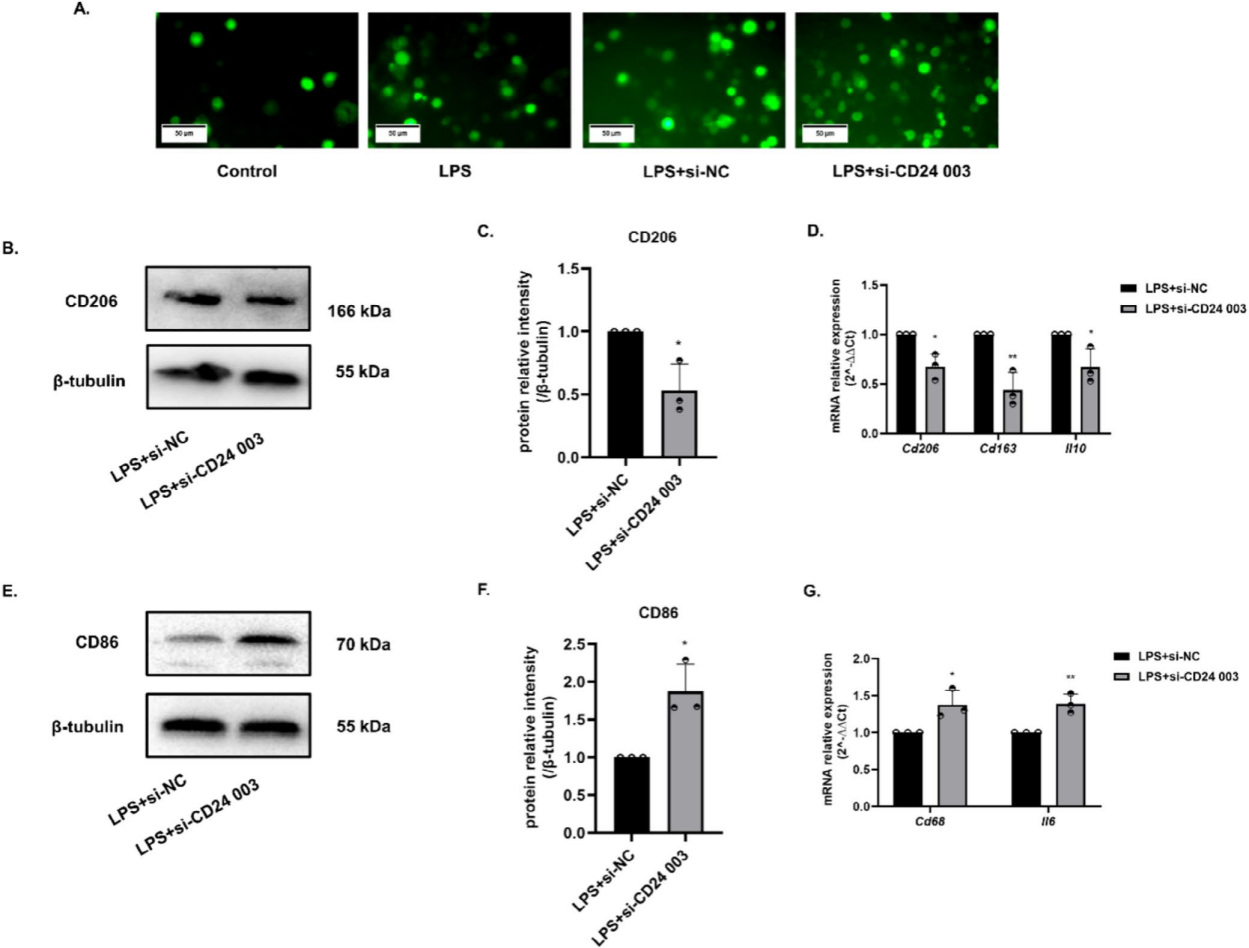
Inhibition of CD24 enhanced oxidative stress in BV2 cells and promoted the expression of M1 microglia. (A) ROS levels were detected by fluorescence. (B, C) The expression level of CD206 was detected by WB. (D) qRT–PCR was used to detect the gene expression levels of *Cd206, Cd163*, and *Il10*. (E, F) WB was used to detect the expression level of CD86. (G) qRT–PCR was used to detect the gene expression levels of *Cd68* and *Il6*. All data were expressed as mean ± SEM (*p < 0.05, **p < 0.01, ***p < 0.001 vs. LPS+ LPS + si‐NC; *t*‐test; *n* = 3).

The results showed that after interfering with the expression of CD24, the oxidative stress level of LPS + si‐CD24 003 group was increased compared with LPS + si‐NC group. Thus, negative regulation of CD24 on cellular oxidative stress level was further demonstrated (Figure 5A). Inhibition of CD24 would increase the expression of CD86, the marker of M1 microglia, as well as the related molecules *Cd68* and *Il6* (Figure 3E–G). In contrast, the expression level of CD206, the marker of M2 microglia, was downregulated, and its related molecules, *Cd163* and *Il10*, also showed a downward trend (Figure 5B–D).

### 3.6 Migration of CD24 in MCAO Mice

**FIGURE 6:**
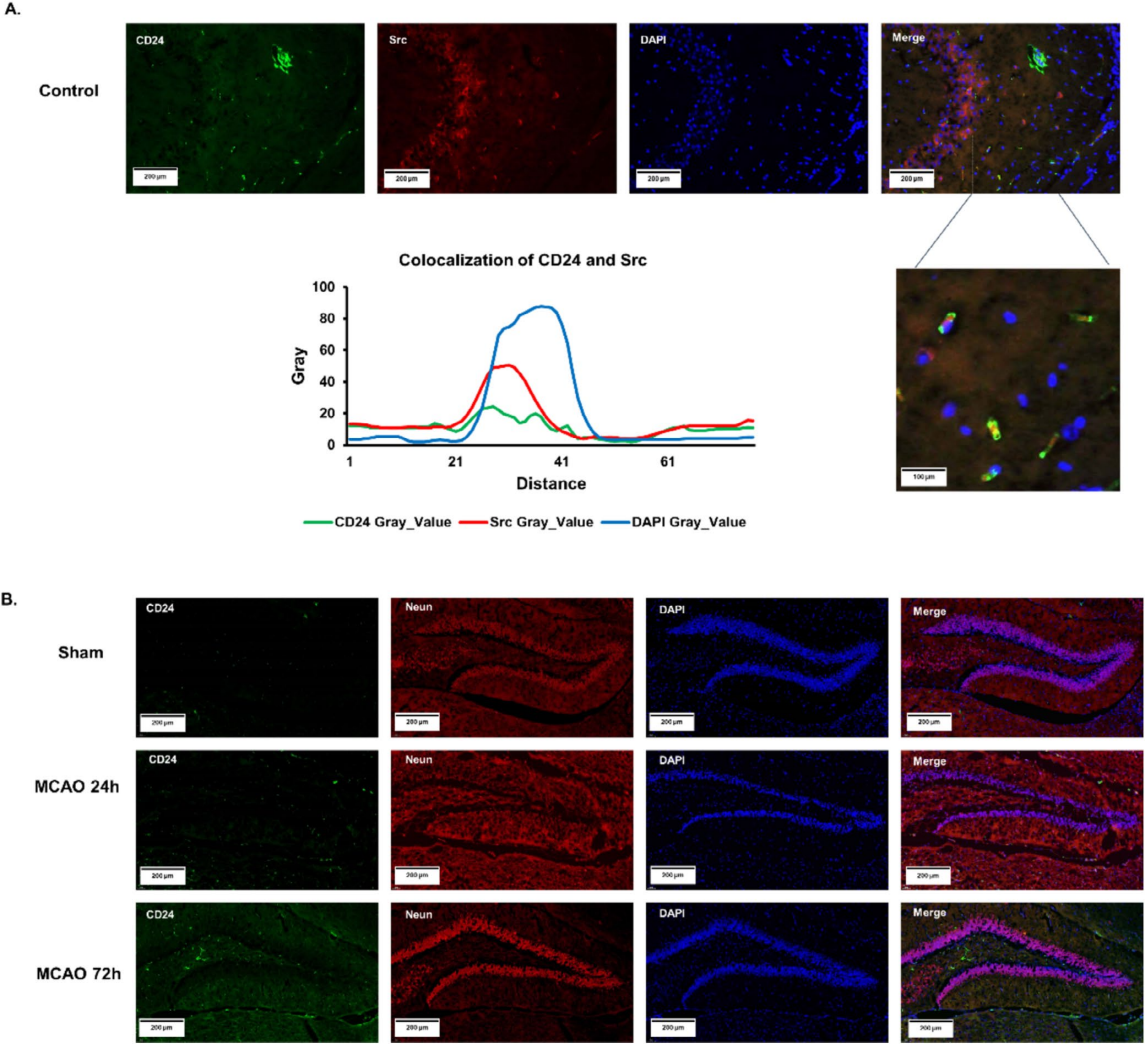
Migration of CD24 in MCAO mice brain. (A) Double immunofluorescent staining of CD24/Src. (B) Double immunofluorescent staining of CD24/Neun.

The double immunofluorescent staining of CD24/Src in the left mouse brain showed obvious signs of merging of CD24 and Src (Figure 6A). The Src‐mediated signaling pathway is related to cell migration. Therefore, we hypothesized that the CD24‐mediated cell migration might be related to Src. Then, the double immunofluorescent staining of CD24/Neun was performed and showed that with the increase of reperfusion time, CD24 that distributed on microglia began to migrate to hippocampal neurons (Figures 1B and 6B). So we speculated that CD24 could regulate the migration ability of microglia cells probably through Src‐mediated migration pathway.

### 3.7 Overexpression of CD24 Increased the Migration Ability of BV2 Cells

**FIGURE 7:**
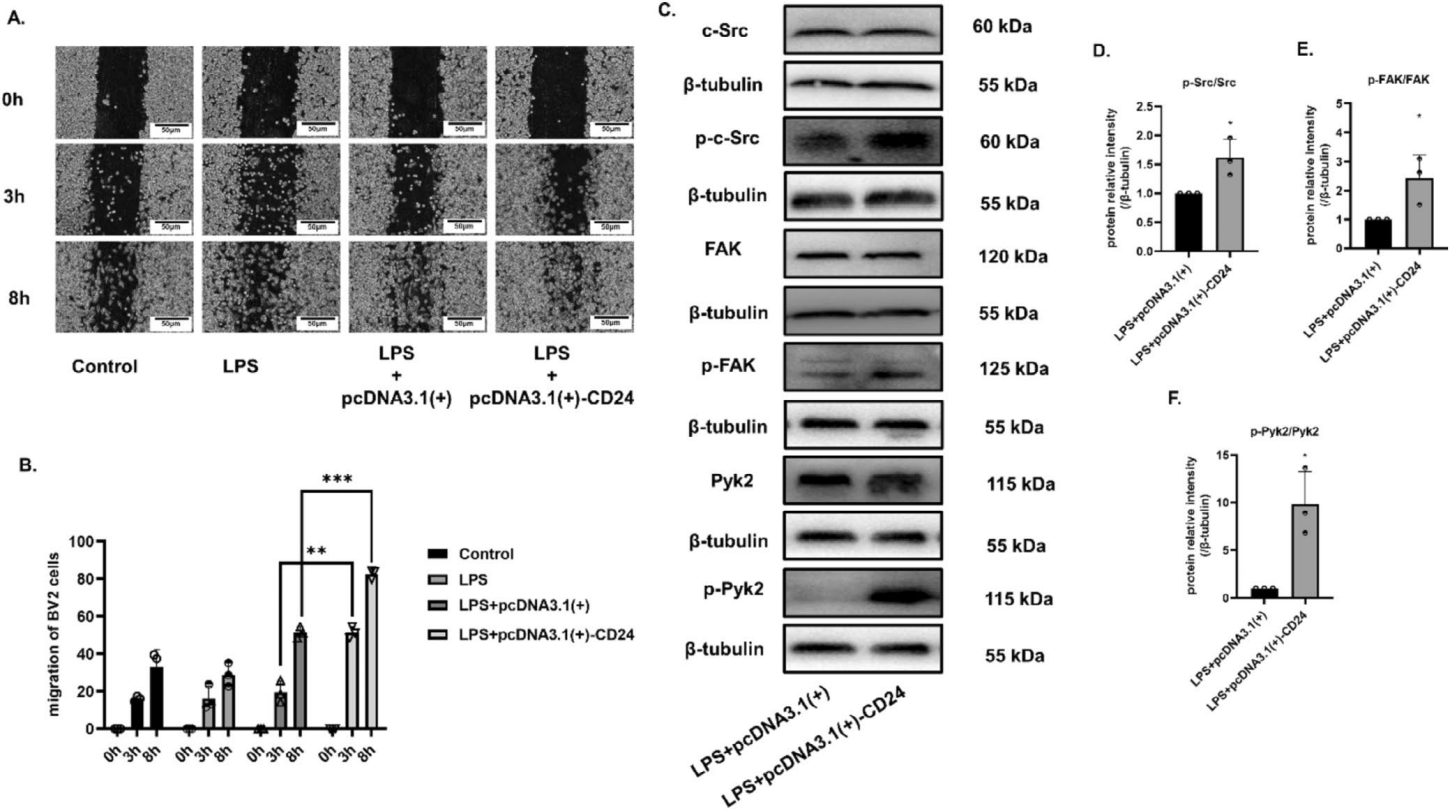
Overexpression of CD24 promoted cell migration via the Src/Fak/Pyk2 migration pathway. (A, B) Cell scratch assay. (C‐F) The phosphorylation levels of c‐Src, Fak, and Pyk2 were detected by WB. All data were expressed as mean ± SEM (*p < 0.05, **p < 0.01, ***p < 0.001 vs. LPS + pcDNA3.1(+); *t*‐test; *n* = 3).

We selected BV2 cells to explore the mechanism of CD24‐mediated migration. The results of the cell scratch trial showed that overexpression of CD24 could significantly improve the migration ability of microglia cells (Figure 7A,B). Fak and Pyk2 are downstream signaling molecules of Src and are related to migration. The results of WB proved that the phosphorylation levels of Src, Fak and Pyk2 were significantly increased after CD24 overexpression, which promoted the migration ability of microglia cells (Figure 7C–F).

### 3.8 Inhibition of CD24 Decreased the Migration Ability of BV2 Cells

**FIGURE 8:**
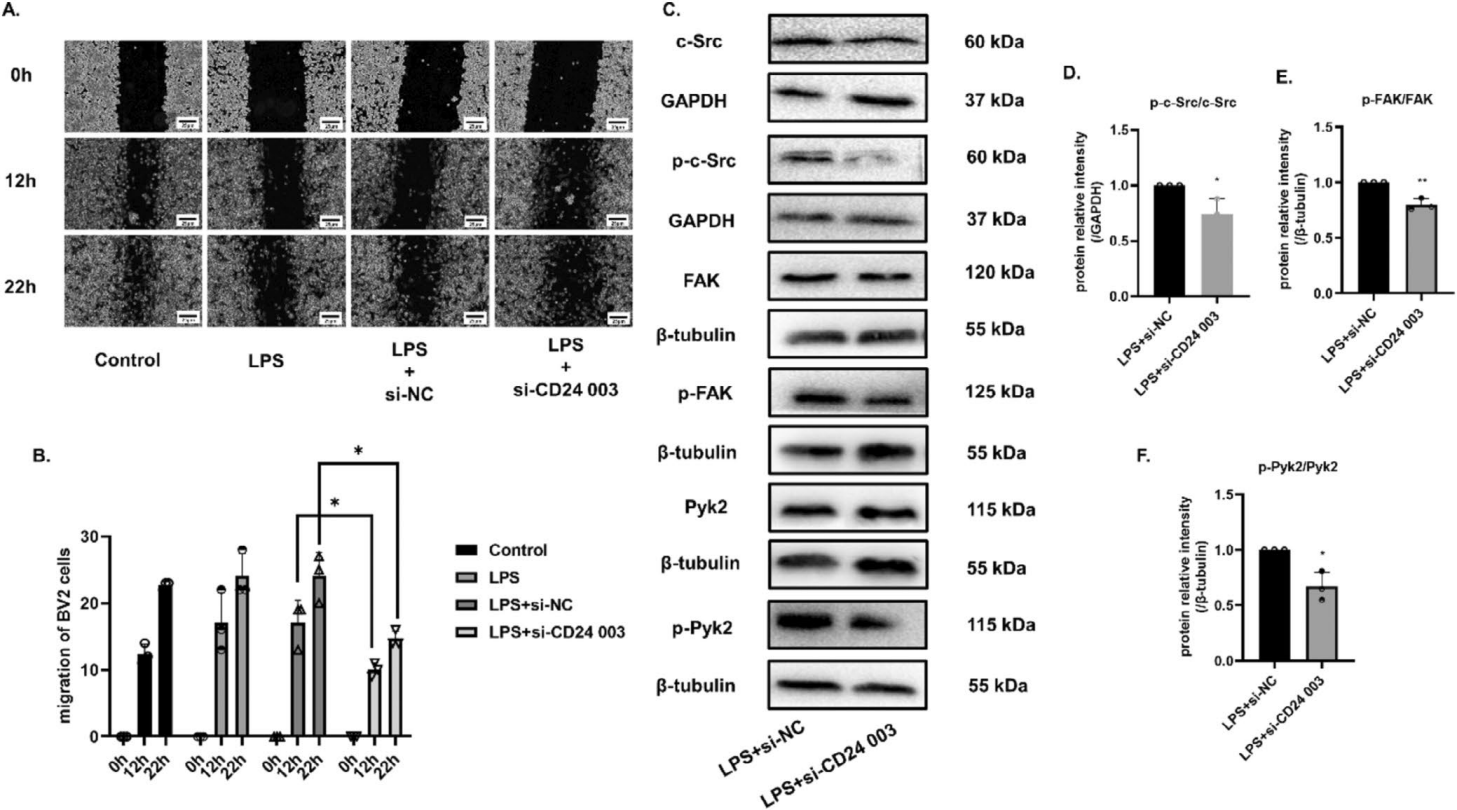
Inhibition of CD24 decreased cell migration via the Src/Fak/Pyk2 migration signaling pathway. (A, B) Cell scratch assay. (C–F) The phosphorylation levels of c‐Src, Fak, and Pyk2 were detected by WB. All data were expressed as mean ± SEM (*p < 0.05, **p < 0.01, ***p < 0.001 vs. LPS + LPS + si‐NC; *t*‐test; *n* = 3).

On the reverse side, we testified that the migration ability of cells was significantly weakened after CD24 interference on the cell scratch trail (Figure 8A,B). Accordingly, the phosphorylation levels of Src, Fak, and Pyk2 all showed downward trends (Figure 8C–F).

### 3.9 SPRC Increased the Expression of CD24 in BV2 Cells in vitro

**FIGURE 9:**
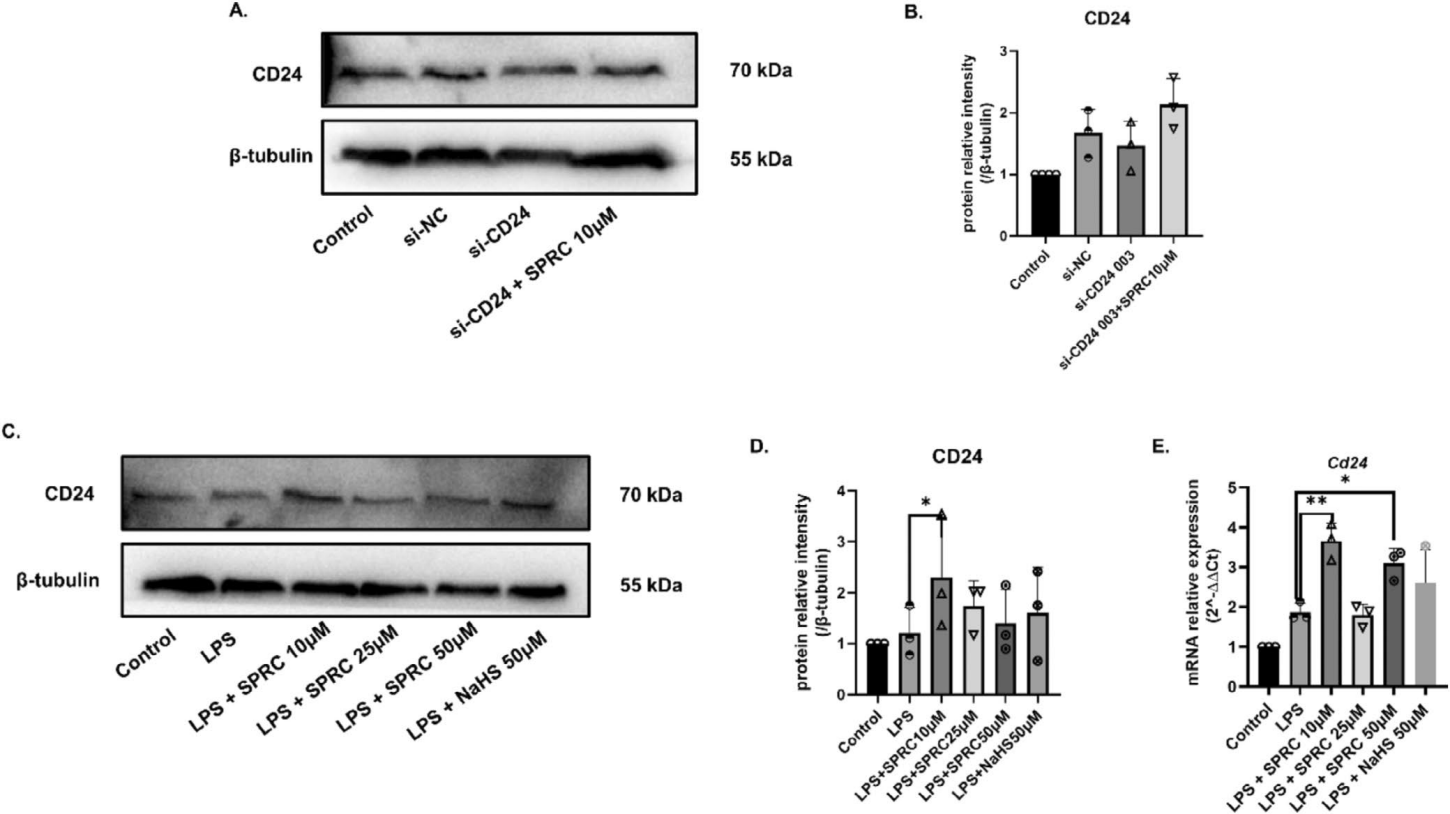
SPRC increased the expression of CD24 in BV2 cells in vitro. (A, B) The protein expression level of CD24 was detected by WB. (C–E) Effects of different doses of SPRC on regulating CD24 in BV2 cells. The protein expression level of CD24 was detected by WB and qRT–PCR. All data were expressed as mean ± SEM (*p < 0.05, **p < 0.01, ***p < 0.001 vs. LPS; one‐way ANOVA; *n* = 3).

To preliminarily explore whether SPRC can play a role in treating ischemic stroke by regulating CD24, BV2 cells were selected for in vitro trials. The experimental results showed that after inhibiting the expression of CD24 in BV2 cells, 10 μM SPRC could effectively reverse the expression of CD24 (Figure 9A,B). Then, LPS was used to create the model for 24 h and treated with different concentrations of SPRC (10 μM, 25 μM, 50 μM) and the positive reagent NaHS (50 μM) for 4 h. The protein and gene expression levels of CD24 were detected by WB and qRT–PCR. The results showed that 10 μM SPRC had the best significant upregulation of CD24 expression (Figure 9C,D). Therefore, we speculated that SPRC could alleviate the level of neuroinflammation by upregulating the expression CD24.

### 3.10 SPRC Decreased Infarct Size and CD24 Expression in MCAO Mice

**FIGURE 10:**
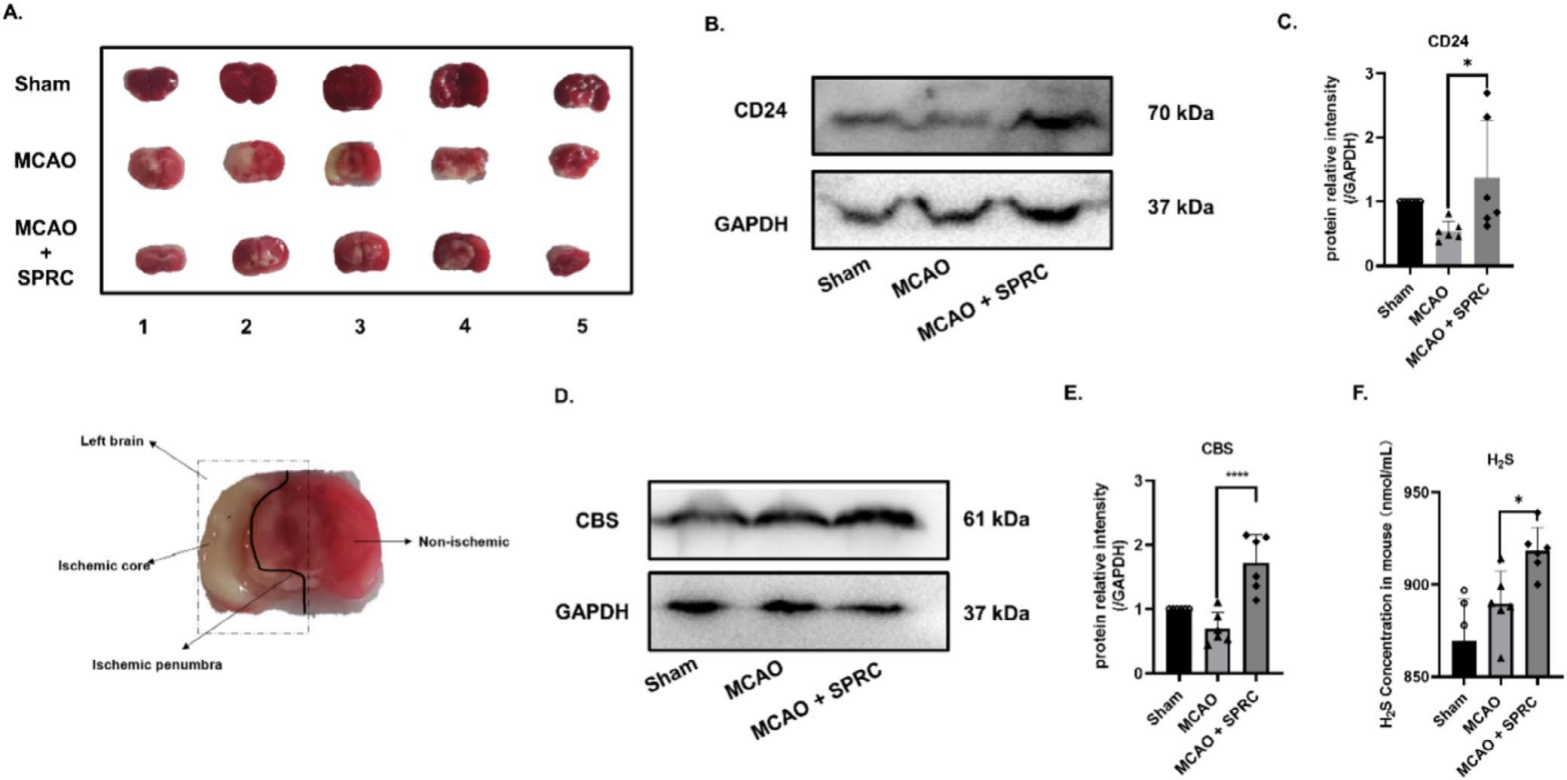
SPRC decreased infarct size and CD24 expression in MCAO mice. (A) TTC was used to detect the cerebral infarction area. (B, C) WB was used to detect CD24 protein expression. (D, E) CBS was detected by WB. (F) H_2_S detection kit to detect the concentration of H_2_S in serum. All data were expressed as mean ± SEM (*p < 0.05, **p < 0.01, ***p < 0.001 vs. MCAO; one‐way ANOVA, *n* = 6).

Since SPRC has been proven to be able to positively regulate CD24 at the cellular level, in vivo studies were also conducted to comprehensively explore the relationship between SPRC and CD24, as well as the therapeutic effect of SPRC on MCAO mice. The animals were divided into three groups: Sham group (Sham), model group (MCAO) and treatment group (MCAO + SPRC10 mg/kg). The results of TTC staining showed that the infarct size of the left brain was significantly decreased in the treatment group (Figure 10A). Moreover, the WB results showed that the expression of CD24 in the treatment group was significantly higher than that in the model group (Figure 10B,C). Thus, it is confirmed that SPRC can upregulate CD24. But how does SPRC act on CD24? According to the previous researches, the effects of relieving inflammation of SPRC on cardiovascular diseases are related to the level of endogenous H_2_S. Therefore, we hypothesized that H_2_S might be an intermediate molecule between SPRC and CD24.

The results of WB showed that the CBS (an enzyme can catalyze H_2_S) level in the treatment group was higher than that in the model group (Figure 10D,E). The concentration of H_2_S in serum was tested by H_2_S detection kit, and the results showed that H_2_S in the treatment group significantly increased (Figure 10F). Therefore, the mechanism could be owing to the regulation of CBS by SPRC with the promoted expression of H_2_S in brain tissue, and H_2_S further stimulated the expression of CD24 and ultimately alleviated stroke diseases.

### 3.11 SPRC Alleviates Inflammation in MCAO Mice by Regulating CD24 Through H_2_S

**FIGURE 11:**
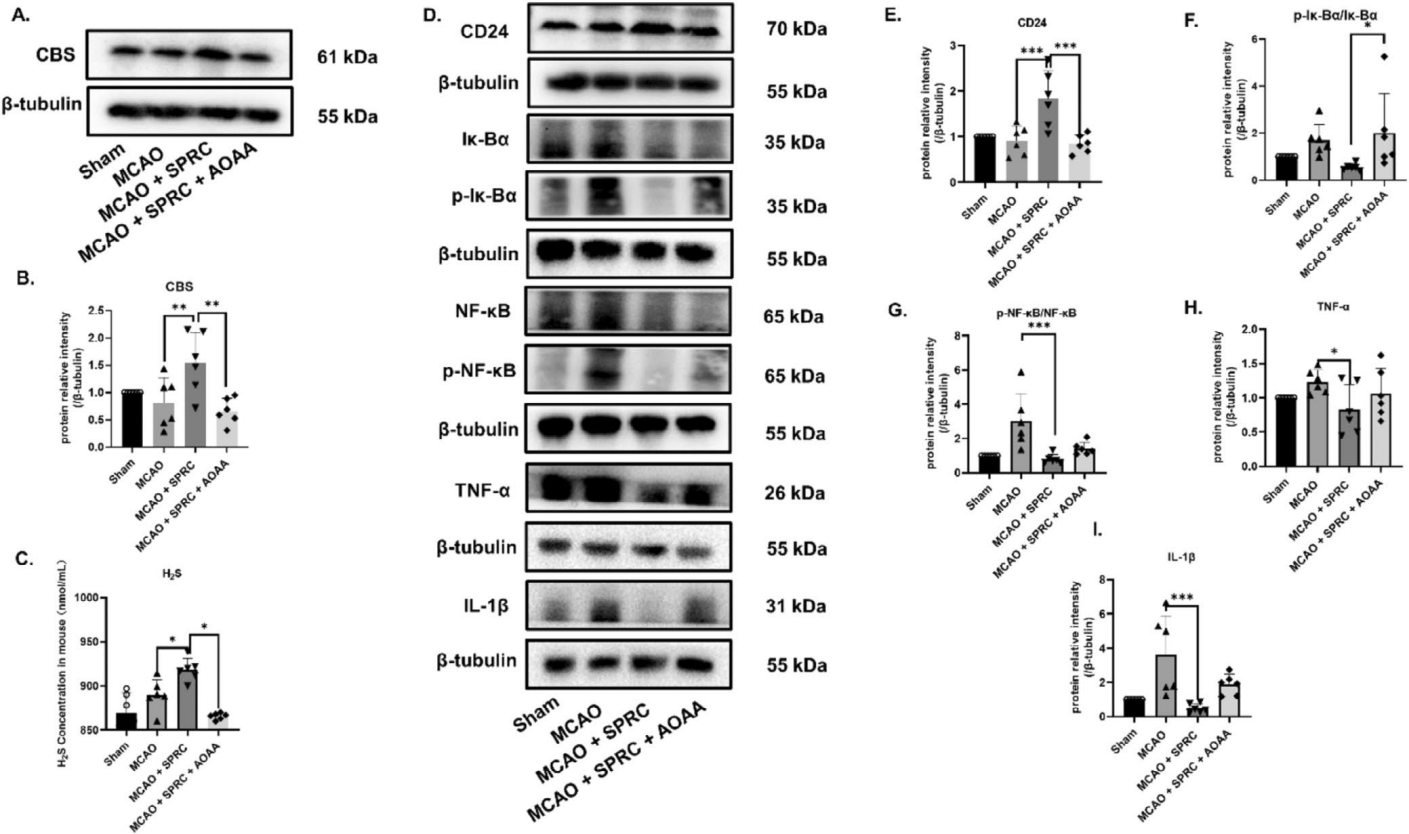
SPRC alleviates inflammation in MCAO mice by regulating H_2_S/CD24. (A, B) The expression of CBS was detected by WB. (C) Detection of H_2_S content in serum. (D–I) CD24 and its inflammatory signaling pathway proteins were detected by WB. All data were expressed as mean ± SEM (*p < 0.05, **p < 0.01, ***p < 0.001 vs. MCAO+SPRC; one‐way ANOVA; *n* = 6).

To further verify the relationship between H_2_S and CD24, animals were divided into four groups: Sham group (Sham), model group (MCAO), Treatment group (MCAO+SPRC) and CBS inhibitor group (MCAO + SPRC + AOAA). It showed that the CBS inhibitor group could significantly reduce the production of CBS enzyme and H_2_S level in vivo compared with the therapeutic group (Figure 11A–C). Simultaneously, the expression of CD24 significantly increased in the treatment group, but significantly decreased in the inhibitor group (Figure 11D,E). This indicated that the concentration of H_2_S could positively regulate the expression of CD24. The CD24‐mediated Iκ‐Bα/NF‐κB inflammatory signaling pathway, as well as the levels of inflammation markers such as TNF‐α and IL‐1β significantly decreased in the treatment group and increased in the CBS inhibitor group (Figure 11D–I). This has further confirmed that SPRC can negatively regulate inflammation through H_2_S/CD24. It can be concluded that the therapeutic mechanism of SPRC is through CBS/H_2_S‐CD24‐Iκ‐Bα‐NF‐κB pathway to relieve inflammation of ischemic stroke.

### 3.12 SPRC Enhances M2 Microglia Migration by Regulating CD24 in MCAO Mice

**FIGURE 12:**
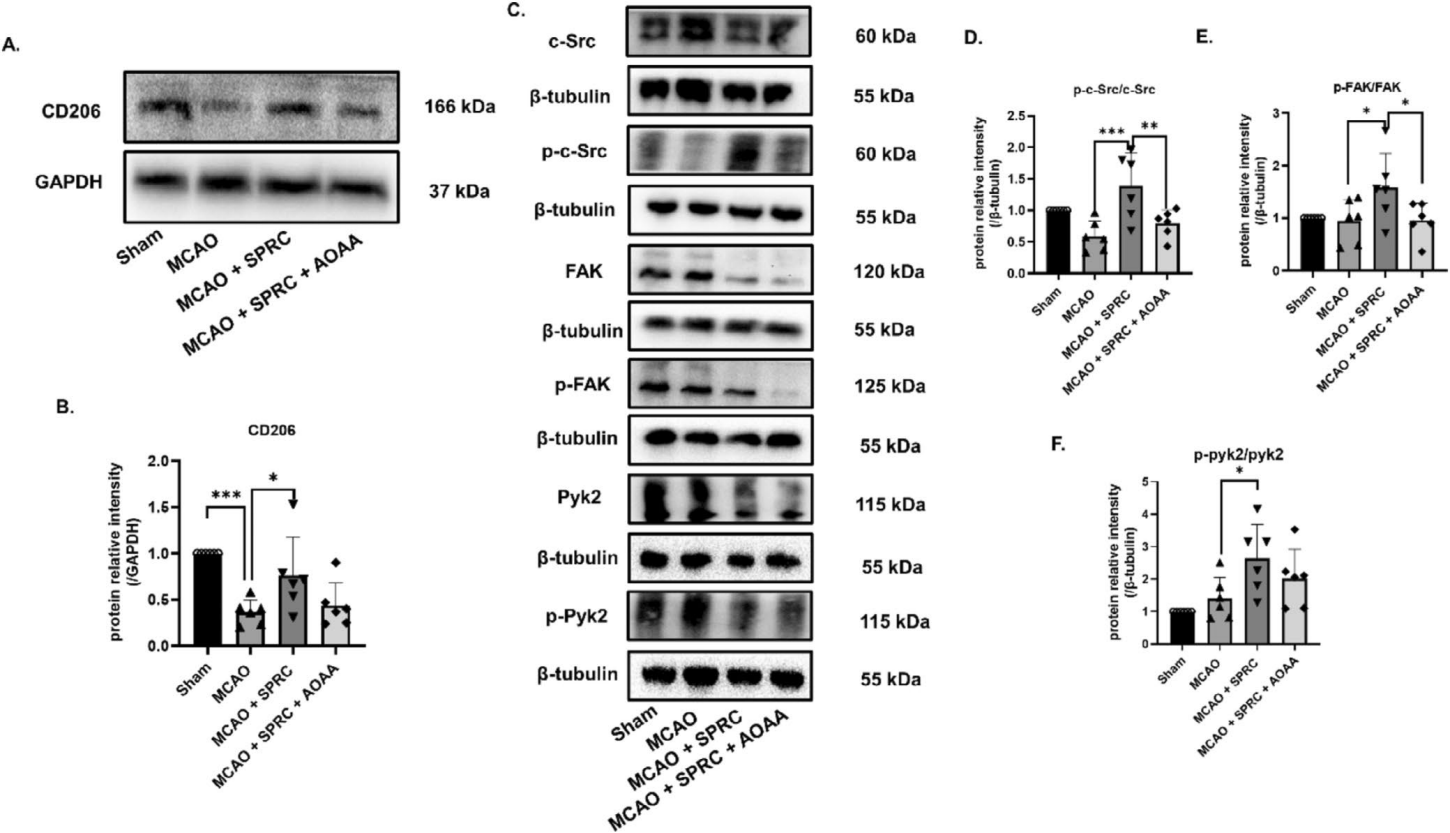
SPRC promoted the migration of M2 microglia in MCAO mice by regulating CD24/Src pathway. (A, B) WB detected the expression of CD206. (C–F) WB was used to detect the phosphorylation levels of c‐Src, Fak, and Pyk2. All data were expressed as mean ± SEM (*p < 0.05, **p < 0.01, ***p < 0.001 vs. MCAO+SPRC; one‐way ANOVA; *n* = 6 animals for group).

Additionally, in the MCAO model, the expression of CD206 in the treatment group was significantly higher than that in the model group (Figure 12A,B). The phosphorylation levels of Src, Fak, and Pyk2 in the treatment group all significantly increased in the SPRC treatment group compared with the model group and CBS inhibitor group (Figure 12C–F). Therefore, SPRC can also promote the migration ability of M2 microglia by regulating CD24/Src/Fak/Pyk2 pathway to alleviate ischemic stroke.

## 4 Discussion

In this study, we have explored innovative new therapies for ischemic stroke and focused on the correlation between neuroinflammation mechanism of ischemic stroke and the immune checkpoint inhibitor CD24. For the first time, we have shown that CD24 is a very promising target for treating ischemic stroke.

LPS is an inducer of neuroinflammation. When LPS binds to TLR of BV2 cells, the downstream signaling pathway is activated through phosphorylating IκB kinase (IKK). The phosphorylation of IKK further mediates the degradation of IκB, thereby activating the NF‐κB signaling pathway [34], then promoting gene transcription and expression [35, 36, 37], such as TNF‐α, IL‐1β, IL‐6, IFN‐γ, etc. [38, 39]. Studies have found that inhibiting NF‐κB phosphorylation could attenuate inflammation [40, 41, 42]. CD24 could ease the inflammatory response by inhibiting the transcriptional activity of NF‐κB after TBI [19]. Consistent with these studies, we firstly showed that CD24 was distributed in microglia cells and CD24 could negatively regulate the expression of inflammatory TNF‐α and IL‐1β after MCAO (Figure 1). In neuroinflammatory model of BV2 cells induced by LPS, since the main objective of this study is to explore the function of CD24, to avoid the influence brought by the plasmid, we simply used LPS + si‐NC/pcDNA(+) and LPS + si‐CD24/pcDNA(+)‐CD24 to compare the data. In the i*n vitro* studies, we further demonstrated that CD24 could negatively regulate the classical signaling pathway of NF‐κB and downregulate the expression of inflammatory TNF‐α and IL‐1β of BV2 cells (Figure 2 and Figure 4).

Microglia migration is a fundamental function during microglia activation. Studies have reported that Src kinase is key to regulate cell proliferation, differentiation, migration, invasion, and angiogenesis [43]. Eyvazi et al. [44] have reviewed that CD24 dysregulates different signaling pathways in various cancer cells, including Src kinases, STAT3, etc. In our study, we found that CD24 and Src overlapped significantly in mice brain, suggesting that CD24 might play a role in the migration of microglia cells (Figure 6). Fak and Pyk2 are also closely related to cell migration and invasion [45, 46]. Studies have shown that the inhibition of Src‐Fak pathway can reduce the invasion, adhesion and migration of tumor cells [47, 48, 49, 50, 51, 52, 53, 54, 55]. What's more, referring to the previous studies in our lab, we have confirmed that the Src/Fak/Pyk signaling pathway can mediate macrophage migration in myocardial infarction [55, 56]. Our results also have showed that CD24 expression can promote the phosphorylation level of Src/Fak/Pyk2 to enhance the migration ability of BV2 cells (Figure 7 and Figure 8). The current study mainly focused on how CD24 regulated the expression of Src/Fak/Pyk signaling protein. However, further activating the Src/Fak/Pyk signaling pathway and NF‐kB signaling pathway via genetic or pharmacological strategies in CD24 overexpression condition can be helpful to reveal the regulating mechanism in depth.

The polarization of microglia is closely related to neuroinflammation. The activated microglia were divided into M1 pro‐inflammatory type and M2 anti‐inflammatory type. It has been shown that M1 microglia promote secondary brain damage, whereas M2 microglia facilitate recovery after ischemic stroke [57, 58, 59]. To explore the effect of CD24 on the polarization of BV2 cells, we detected the markers of different polarization of BV2 cells. The results preliminarily found that the up‐expression of CD24 can promote M2 polarization of microglia (Figure 3B–G and Figure 5B–G), which suggested that CD24 could reduce cell inflammation by regulating the polarization of microglia. But how does CD24 affect the polarization of BV2 cells needs to be further explored. Furthermore, anti‐inflammatory M2 microglia has a certain repair effect on neuron damage, but whether this repair effect is related to CD24 is worthy to be explored.

All the results of these studies have demonstrated that CD24 could negatively regulate neuroinflammation and promote the migration of M2 in BV2 cells, suggesting that CD24 is potentially an innovative therapeutic target of ischemic stroke. Based on the above mechanism study, for the first time, we explored the efficacy of SPRC in ischemic stroke and the connection between SPRC and CD24. Firstly, in vitro, we found SPRC could positively regulate the expression of CD24 (Figure 9). As the preliminarily observation on whether SPRC can alter the expression of CD24, according to the former studies, the most common dosages of SPRC, 10 μM, 25 μM, and 50 μM have been used. However, only 10 μM of SPRC could promote the expression of CD24. The dose‐independent influence of SPRC on CD24 could be due to the indirect regulation of SPRC via CBS enzymes to produce H_2_S, which then regulates CD24. If CBS is up to saturation, the dose‐dependent of SPRC on CD24 could disappear because the amount of H_2_S produced might be limited. Though not in a dose‐dependent manner, the relationship between CD24 and SPRC has been confirmed. It is interesting to further study how SPRC indirectly regulates CD24 and what is the optimized dose of SPRC to increase CD24 level. Meanwhile, this phenomenon also has been observed in MCAO mice. SPRC can decrease infarct size of the brain after MCAO and can upregulate the expression of CD24 (Figure 10). But how SPRC regulates CD24 is unknown. Studies have shown that SPRC has good effects on relieving inflammation on cardiovascular disease and autoimmune disease through elevating H_2_S level by mainly modulating CSE/H_2_S pathway [28, 29, 30, 31]. But in brain, CBS is the dominant enzyme that produces endogenous H_2_S. To further explore the association with SPRC, H_2_S, CBS, and CD24, we used AOAA to inhibit the production of CBS, thereby reducing the release of endogenous H_2_S. All data revealed that the connection between SPRC and CD24 is indirect. SPRC was proved to upregulate the expression of CD24 via CBS/H_2_S. But whether the regulation of H_2_S on CD24 is direct or not remains to be further studied.

In our study, to explore the function of CD24 in ischemic stroke more quickly, we chose to use siRNA to knockdown CD24 instead of using knockout mice. We observed the chain reactions resulting from a 50% knockdown efficiency of the expression of CD24 in the in vitro trails, but it is also essential to establish a consistent knockdown‐CD24 MCAO model in future studies. In the view of these, we finally revealed that the new mechanism of ischemic stroke treatment of SPRC is associated with the CBS/H_2_S/CD24/Iκ‐Bα/NF‐κB inflammatory pathway (Figure 11) and CBS/H_2_S/CD24/Src/Fak/Pyk2 migration pathway (Figure 12).

## 5 Conclusions

In conclusion, this study shows that CD24 may be a potential innovative target of ischemic stroke for alleviating neuroinflammation via NF‐κB pathway and Src pathway. The treatment of SPRC on ischemic stroke is associated with CD24 via CBS/H_2_S pathway. This research has revealed a potential therapy for neuroinflammation in ischemic stroke.

## Author Contributions

Chenye Wang designed the studies, performed experiments, analyzed the data, and wrote the manuscript. Sha Li performed experiments. Haiyan Xi and Qixiu Li revised the manuscript. Jiejia Li, Qing Zhu supported the study. Pinwen Wu, Yi‐Zhun Zhu and Yicheng Mao supervised the study and revised the manuscript. All authors have approved the manuscript.

## Conflicts of Interest

The authors declare no conflicts of interest.

## Data Availability

The data that support the findings of this study are available from the corresponding author upon reasonable request.
